# Influences of Friedel’s Salt Produced by CaO-Activated Titanium-Extracted Tailing Slag on Chloride Binding

**DOI:** 10.3390/ma16072843

**Published:** 2023-04-03

**Authors:** Song Tang, Tongjiang Peng, Hongjuan Sun, Wenjin Ding, Liming Luo, Hao You, Xiaoman Yao

**Affiliations:** 1 School of Environment and Resources, Southwest University of Science and Technology, Mianyang 621010, China; 2 Institute of Mineral Materials and Application, Southwest University of Science and Technology, Mianyang 621010, China; 3 Key Laboratory of Solid Waste Treatment and Resource Recycle, Ministry of Education, Mianyang 621010, China

**Keywords:** chlorine-containing industrial waste, titanium-extracted tailing slag, alkali-activated, CaO, Friedel’s salt, chloride binding, strength

## Abstract

Titanium-extracted tailing slag (TETS) has high activity, but the content of chloride ions is high. To effectively bind the chloride ions, CaO was used to activate the TETS, and the solidified cementitious material of CaO-activated TETS was prepared. The effects of CaO content and curing age on the strength of solidified samples, chloride binding capacity, and chloride binding mechanism were studied. By means of XRD, FTIR, SEM, and EDS, the hydration reaction products, microstructure, morphology, and micro-components of the solidified sample were characterized. The results show that the chloride ions can be effectively bound by using CaO to activate TETS with higher mechanical strength. When the CaO content is 10 wt%, the strength of the 28-day-cured body can reach more than 20 MPa, the chloride ion binding amount is 38.93 mg/g, and the chloride binding rate is as high as 68%. The new product phases of the solidified sample are mainly Friedel’s salt (FS) and calcite, in which the amount of FS production and the degree of crystal development are affected by the CaO content and curing age. The chloride binding ions in the solidified sample are mainly the chemical binding by FS. The FS diffraction peak strength increases with the increase of CaO content and curing age, but the calcite diffraction peak strength is less affected by them. FS mainly accumulates and grows in the pores of the solidified sample. It can optimize the pore structure of the solidified sample and improve the strength of the solidified sample while binding chloride ions. The results can provide useful information for the resource utilization of chlorine-containing TETS, the improvement of durability of Marine concrete, and the application of sea sand in concrete.

## 1. Introduction

Panzhihua, Sichuan Province, China, is rich in titanium resources, among which TiO_2_ reserves reach 130 million tons, accounting for about 1/3 of the world’s proven titanium reserves [1]. Under the current smelting process conditions, about 50% of titanium in raw ore enters smelting slag, forming titanium-bearing blast furnace slag. At present, more than 70 million tons have been piled up, and the rate is still increasing by more than 3 million tons every year [2]. Local enterprises adopt a high-temperature carbonization–low-temperature selective chlorination method to remove titanium from titanium-bearing blast furnace slag and have achieved good results. However, a kind of industrial solid waste—TETS—is obtained after titanium removal [3]. TETS has high chemical reactivity [4], but it usually contains about 2~7 wt% of chloride ions, so it is difficult to be used in cement and concrete admixture. It can only be stored. While occupying a large amount of land resources, the stored TETS also poses potential hazards to the surrounding environment. It has become a key factor to restrict the sustainable development of this titanium extraction process.

It is common to produce structural materials using solid wastes [5,6,7]. Due to the high chlorine ion content in TETS, the chloride binding capacity of the gelling has become the focus of attention when it is applied as a resource, but there is rarely any study on the binding of chloride ions in TETS until now.

Chloride ion is a main factor influencing durability of concrete structures [8,9,10]. Chloride exists in two major states in concrete, namely, the free state and the binding state [11,12]. Free-state chloride is the major cause of corrosion of steel bars, while binding-state chloride is harmless to steel bars and the surrounding environment [13]. The results prove that the chloride ions in the gelled material can be bound by 40~60%. Chloride binding includes physical binding and chemical binding [14,15,16,17]. Among them, physical binding mainly refers to the physical adsorption of chloride ions by calcium silicate hydrate gel in cementing materials [18], while chemical binding refers to the chemical combination of chloride ions into some insoluble salts such as Kuzel’s salt and FS. The latter is most common [19,20].

FS is the main hydration product of chemically binding chloride ions, with a flake crystal structure [21]. Increasing the FS content in structural materials is helpful to improve the chemical binding ability of chloride ions, and improving the chemical binding capacity of gelling materials is an effective way to enhance the chloride binding capacity [22]. Studies have shown that the chloride binding capacity of FS in cementing materials is about five times that of C-S-H gel [23]. The content of FS sometimes becomes an important index to measure the chloride binding capacity of cementing materials [24].

In the study of chloride binding in cementitious materials, the internal chloride ions are mainly realized by using sea sand, seawater, or adding chloride salts such as sodium chloride, calcium chloride, and magnesium chloride [25,26,27,28,29]. The TETS contains soluble chloride salts such as CaCl_2_ and MgCl_2_, which have a high content of chloride ions and a complex existence status. Therefore, the study on chloride binding in TETS is not only helpful to promote the resource utilization of it, but also has important reference significance for the application of sea sand in concrete, the durability of concrete, and the study on the solidification ability of chloride ions.

The main chemical components of TETS are CaO, SiO_2_, and Al_2_O_3_. It does not have a self-gelling property. When alkali activation was used in TETS, it showed higher chemical activity [4,30]. Among many alkali activators, CaO has the advantages of safety, environmental protection, easy access to products, and low price, and CaO is the main component of TETS. Using CaO as activator will not increase the new element, but it will increase the content of Ca^2+^ in the solidified sample. Increasing the content of Ca^2+^ will help to increase the content of FS in the solidified sample and the ability of C-S-H gel to bind Cl^-^ [31,32].

In this study, CaO was used to activate TETS to produce a solidified material. Moreover, the effects of CaO content and curing age on the strength and chloride binding effect of solidified samples were investigated. The chloride binding mechanism in solidified samples of CaO-activated TETS was disclosed through X-ray diffraction (XRD), scanning electron microscope (SEM), and energy-dispersive spectroscopy (EDS).

## 2. Materials and Methods

### 2.1. Materials

**Figure 1:**
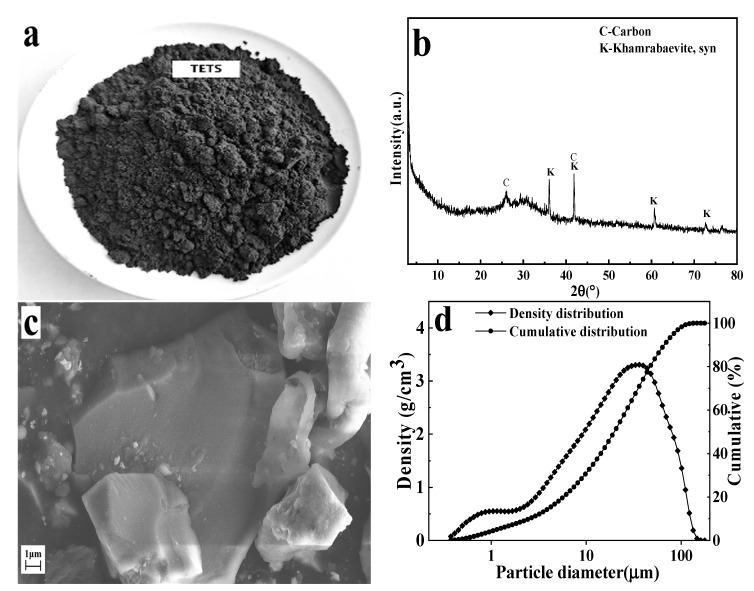
The raw material photograph (**a**), XRD patterns (**b**), SEM images (**c**), and particle size distribution (**d**) of TETS.

The raw materials used in this study were TETS, CaO, and deionized water. TETS was supported by Panzhihua Iron and Steel Research Institute Co., Ltd. (Panzhihua, China). Analytical-grade CaO was produced by Sichuan Chengdu Colon Chemical Co., Ltd. (Chengdu, China). Deionized water was prepared in the laboratory. The chemical composition and loss on ignition of TETS are presented in Table 1. The raw material photograph (Figure 1a), XRD spectra, (Figure 1b), SEM images (Figure 1c), and particle size distribution (Figure 1d) are shown in Figure 1.

As can be seen from Figure 1 and Table 1, TETS is black sand-like particles (Figure 1a), with the main chemical components including CaO, SiO_2_, Al_2_O_3_, TiO_2_, MgO, and Cl, and a small amount of Fe_2_O_3_, SO_3_, and other trace components (Table 1). It is mainly composed of amorphous vitreous, containing a small amount of Khamrabaevite, syn (2θ = ~42.0°), and graphitic carbon (2θ = ~26.3°) (Figure 1b). Its vitreous structure is dense (Figure 1c), and its particle size distribution ranges from 1 to 80 μm, d_50_ = 21.76 μm (Figure 1d).

### 2.2. Experimental and Program

The mass percentage of CaO was selected at 4 wt%, 6 wt%, 8 wt%, and 10 wt%, respectively. The liquid–solid ratio (w/s) was fixed at 0.35. Material compositions of samples are listed in Table 2. TETS and CaO were accurately weighted and poured into a cement mortar mixer, which was slowly stirred for 3 min. Water was added and then quickly stirred for 5 min. The fresh paste, which was evenly mixed, was poured into a plastic bag and sealed for 5 h, followed by molding, curing, and strength test. All tests were referring to Chinese National Standard GB/T 17671-2021. The mortars were set at 4 × 4 × 16 cm and there were 3 specimens in each group. Molded specimens were put into the standard curing room with molds, and they were de-molded after 24 h. Next, the de-molded specimens were put in the standard curing room where the curing temperature and humidity were 20 ± 2 °C and ≥95%. UCS tests were carried out at the curing age of 1, 3, 7, and 28 days.

Broken solidified samples after UCS tests were immersed in acetone for 72 h to terminate hydration. Later, samples were dried in a 50 °C vacuum drying oven for 48 h [33] and appropriate sheet samples were collected for the SEM-EDS test. Appropriate samples were collected and ground for the leaching test, XRD, and the FTIR test. Specifically, soluble chloride ions were leached via the ASTM C1218 method and total chloride ion was leached by ASTM C1152 and ASTM C114 methods. After the leaching solution was diluted, the chloride ion concentration was tested through ion chromatography, based on which the chloride ion concentration of the leaching solution was calculated.

The mass of the chloride binding amount, *Cl_m,b_*, in the dry solidified sample was calculated according to Equation (1):

(1) $$Cl_{m,b}=\left[\frac{1000\times C_{Cl,a}\times n_{a}\times V_{Cl,a}}{M_{s,a}}-\frac{1000\times C_{Cl,w}\times n_{w}\times V_{Cl,w}}{M_{s,w}}\right]\times \rho_{w,t}$$

where *Cl_m,b_* is the chloride binding amount in the dry solidified sample (mg/g), *C_Cl,a_* is the chloride ion concentration of the acid leaching solution of the solidified sample tested by ion chromatography (ppm), *n_a_* is the dilution times of chlorine ions tested by acid leaching solution, *V_Cl,a_* is the total volume of the leaching solution obtained from the acid leaching experiment (mL), and *M_s,a_* is the mass of the dry solidified sample used in the acid leaching experiments (g). *C_Cl,w_* is the chloride ion concentration of the water leaching solution of the solidified sample tested by ion chromatography (ppm), *n_w_* is the dilution times of chlorine ions tested by the water leaching solution, *V_Cl,w_* is the total volume of the leaching solution obtained from the water leaching experiment (mL), *M_s,w_* is the mass of the dry solidified sample used in the acid leaching experiments (g), and $\rho_{w,t}$ is the density of the water at the temperature of the ion chromatography test.

### 2.3. Characterization of Solidified Samples

An Axios-type X-ray fluorescence spectroscope (Panaco, the Netherlands) was used to test chemical composition of TETS. Phase compositions of the used raw materials and solidified samples were analyzed by a JL-1500-type laser particle analyzer (Light Industry Institute, Sichuan) and D/MAX-IIIB-type X-ray diffractometer (Nishiku, Japan), during which parameters were set as follows: Cu target, tube voltage: 40 kV, tube current: 40 mA, power: 2.2 kW, and scanning range: 3~80°. Infrared spectra were collected by an INVENIO S-type infrared spectrometer. The wavenumber range was: 4000–400 cm^−1^, resolution: >0.4 cm^−1^, and wavenumber precision: >0.0005 cm^−1^. The morphology of the paste was tested by the Sigma300 field scanning electron microscope (ZEISS, Germany). Micro-area composition of the solidified sample was tested by the X-MAXN20 EDS (OXFORD, UK). Compressive strength of solidified samples was tested by ETM305F-2 press machine (Shenzhen, China), and the loading rate was 2400 N/s ± 200 N/s. Chloride content in filtrate was tested by IC-881 ion chromatography (Vanstone, Switzerland).

## 3. Results and Discussion

### 3.1. Chloride Binding Content and UCS of Solidified Samples

**Figure 2:**
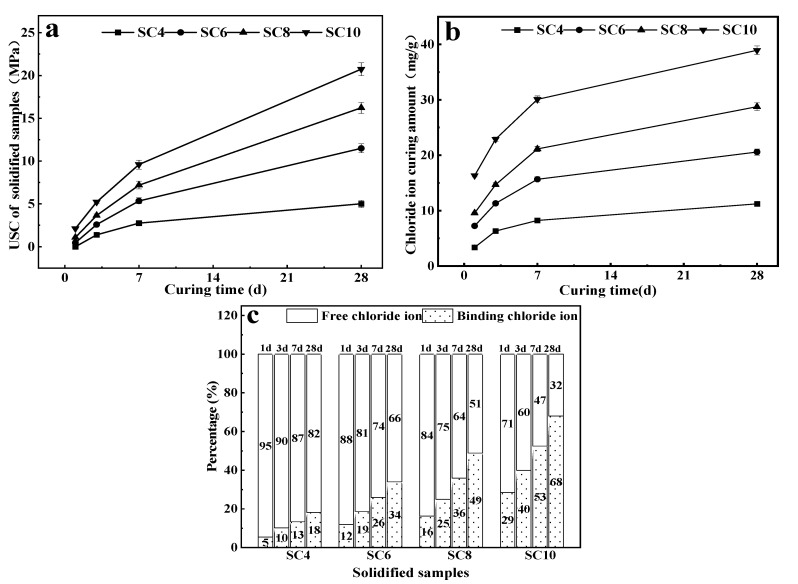
USC (**a**), chloride binding amount (**b**), and chloride binding rate (**c**) of solidified samples with different CaO contents and different curing times.

The unconfined compressive strengths of solidified samples with different CaO contents at different curing ages are shown in Figure 2a. Chloride binding contents are shown in Figure 2b and Table 3. Chloride binding rates are shown in Figure 2c. It can be seen from Figure 2a that SC4 had no obvious strength at 1 day, whereas the strengths of SC6, SC8, and SC10 at 1 day were 0.46 MPa, 1.08 MPa, and 2.12 MPa, respectively. However, studies have shown that when activating GGBS with a CaO content of 6 wt%, 8 wt%, and 10 wt%, the hardened paste at 1 day did not show strength [34]. The results indicate that the activity of CaO-activated TETS was higher than that of CaO-activated GGBS in early ages. The strengths of SC10 at 28 days increased by about 9 times to 20.74 MPa. Meanwhile, although the strength of SC4 at 28 days increased to 5.01 MPa, it was only about 1/4 of that of SC10 at the same age. With the increase of the CaO content and curing time, the UCS of solidified samples increased accordingly, but the growth rates were different. The highest growth rate was achieved before 7 days. After 7 days, the growth rate of strength decreased. Specifically, the strength of SC4 was the lowest among all samples at the same age and its growth amplitude was the lowest. This reflects that when CaO content was lower than a certain value, the activation effect of TETS significantly declined.

When the GGBS powder was activated with 6.25 wt% CaO, the strength at 28 days can reach 42 MPa [35], but the d50 of the GGBS was about 10 μm, and it is more expensive. However, different from GGBS, TETS had nearly zero cost, and it had no preprocessing before activation. Even if 10 wt% CaO was used to activate the GGBS with a specific surface area of 450 m^2^/kg, the strength at 28 days was only about 20 MPa [33,34]. The strength is similar to that of CaO-activated TETS, but the material cost of CaO-activated TETS is 2/3 lower than that of CaO-activated GGBS. When 5 wt% NaOH was used to activate 100 wt% TETS, the strength at 28 days was close to 60 MPa [4]. Compared with NaOH, CaO is superior in terms of safety, environmental protection, and low cost. In view of the strength of solidified samples alone, solidified samples of CaO-activated TETS still have extensive practical value and it is completely applicable to engineering projects with low-strength requirements, such as base course or sub-base course of pavement, baking-free bricks, filling materials, etc.

Figure 2b shows that the curve of chloride binding content in solidified samples was similar to that of its unconfined compressive strength, and both increased with the increase of CaO content and curing age. The highest growth rate was achieved within 7 days, indicating that increasing CaO content and curing age is conducive to chloride binding. For solidified samples with different CaO contents, the growth rates of chloride binding content were significantly different. The chloride binding content of SC4 at 1 day was only 3.36 mg/g, and it increased to only 11.22 mg/g at 28 days. The chloride binding content of SC10 at 1 day was 16.32 mg/g, and it reached 38.93 mg/g at 28 days, which was about 3.5 times that of SC4 at 28 days. Moreover, it was higher than the chloride binding content (16.5~22.9 mg/g) of sodium carbonate-activated slag paste with burnt dolomite after being immersed in 1 M NaCl solution for 72 h [36]. It was more than the chloride binding content of cement paste with 9% Nano-Al_2_O_3_ after being immersed in 1 mol/L of chloride ion solution for 2 months [37].

Figure 2c shows the proportions of binding chloride ions and free chloride ions in the solidified sample with different CaO contents and different curing ages. At 1 day, the content of binding chloride ions in the SC4 sample was only 5%, and even at 28 days, the binding chloride ions were no more than 20%. However, for the SC10 sample, the binding chloride ions in the solidified sample reached 29% at 1 day and nearly 70% at 28 days. In addition, the chloride binding rate in the SC6 solidified sample was about twice that of SC4 at the same age. However, when the content of CaO exceeded 6 wt%, the growth trend of the chloride binding rate in the solidified sample slowed down, indicating that the content of calcium oxide was too low. The chloride binding effect will decrease dramatically. When the content of CaO reached 10 wt%, the increase of the chloride binding rate became larger.

### 3.2. Phase Characteristics of Solidified Samples

**Figure 3:**
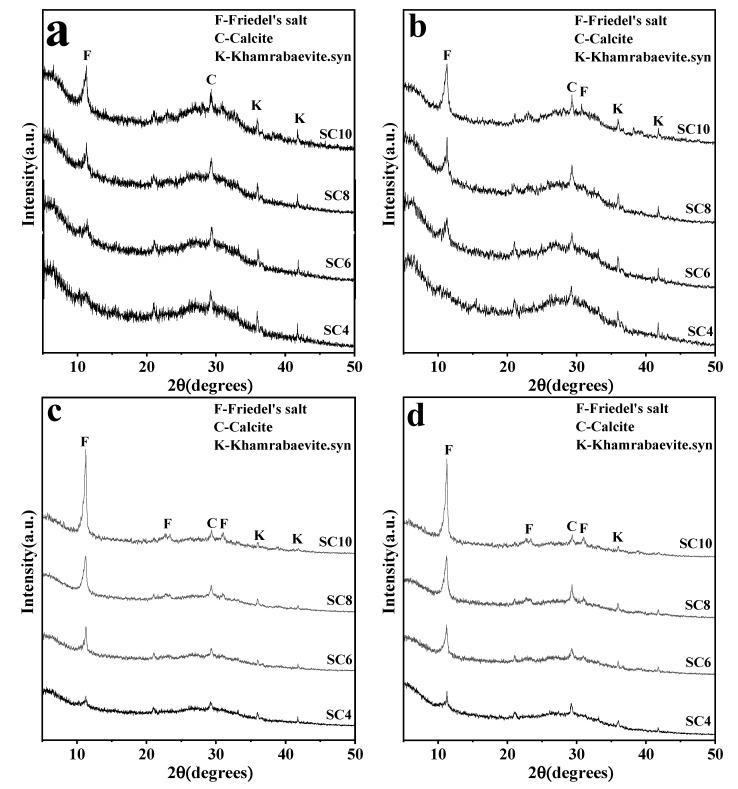
The XRD patterns of solidified samples for: (**a**) 1 day, (**b**) 3 days, (**c**) 7 days, and (**d**) 28 days.

XRD spectra of samples with different CaO contents in the range (5~50°) of 2θ at 1 day (Figure 3a), 3 days (Figure 3b), 7 days (Figure 3c), and 28 days (Figure 3d) are shown in Figure 3. This range covered characteristic peaks of all hydration products in solidified samples. It can be seen from Figure 3 that at 1 day, all solidified samples developed diffraction peaks of the FS (002) crystal surface at 2θ = ~11.3°, except SC4. This is a common hydration product of cement-based binding material or alkali-activated binding material [38,39] when there were chloride ions, and also a major phase of chemical chloride binding. It was until 7 days that SC4 developed a weak diffraction peak of FS, and this diffraction peak was still weak at 28 days. SC10 developed an obvious diffraction peak of FS at 1 day. This proved that CaO content in solidified samples had a great influence on the production of FS, and that increasing CaO content is conducive to the production of FS. When the curing time was extended, the average area of the diffraction peak of FS obviously increased. It was indicated that the development degree of FS crystal became better and better. Increasing the curing age is beneficial to the development of FS crystals.

Except for the diffraction peak of FS, solidified samples with different CaO contents at different curing ages all developed weak diffraction peaks of calcite at 2θ = ~29.3° [33], which was mainly attributed to the reaction between CO_2_ absorbed from air and some Ca(OH)_2_ in solidified samples during the curing period [34].

When CaO was used to activate GGBS, a C-S-H diffraction peak also appeared [35], and a portlandite diffraction peak appeared in early samples [34], but the portlandite diffraction peak did not appear in any age samples of CaO-activated TETS. The reason is that portlandite was produced by hydration reaction of CaO, it quickly reacts with activated alumina and calcium chloride in TETS to form FS, which consumes portlandite in solidified samples in a short time. When CaO was used as the activator, it was reacted with water firstly to produce Ca(OH)_2_ (Equation (2)). The small amount of Ca(OH)_2_ in solidified samples absorbs CO_2_ from the air during the curing period to produce calcite (Equation (3)). Most Ca(OH)_2_ reacted with calcium chloride and activated aluminum oxide in TETS to produce FS (Equation (4)). FS was the new major phase produced in solidified samples and the major hydration product in chemical chloride binding.

CaO + H_2_O = Ca (OH)_2_ (2)

Ca(OH)_2_ + CO_2_ = CaCO_3_↓ + H_2_O (3)

3Ca(OH)_2_ + Al_2_O_3_ + CaCl_2_ + 7H_2_O = 2Ca_2_Al(OH)_6_Cl(H_2_O)_2_↓ (4)

### 3.3. Infrared Spectral Characteristics of Hardened Body

**Figure 4:**
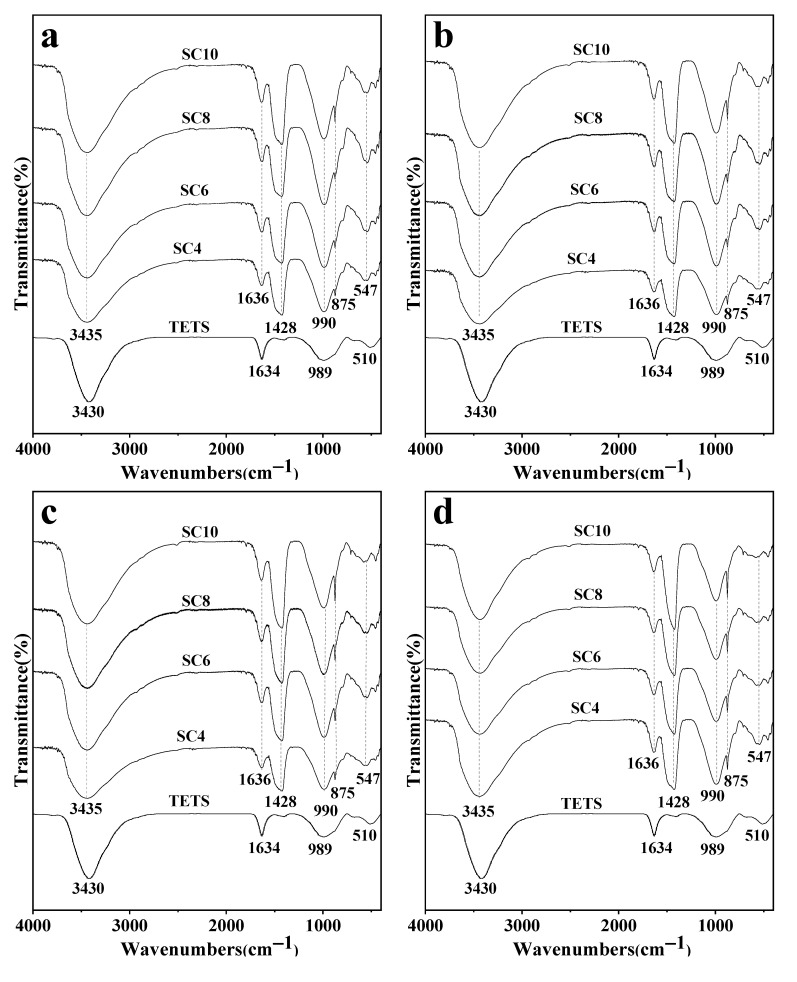

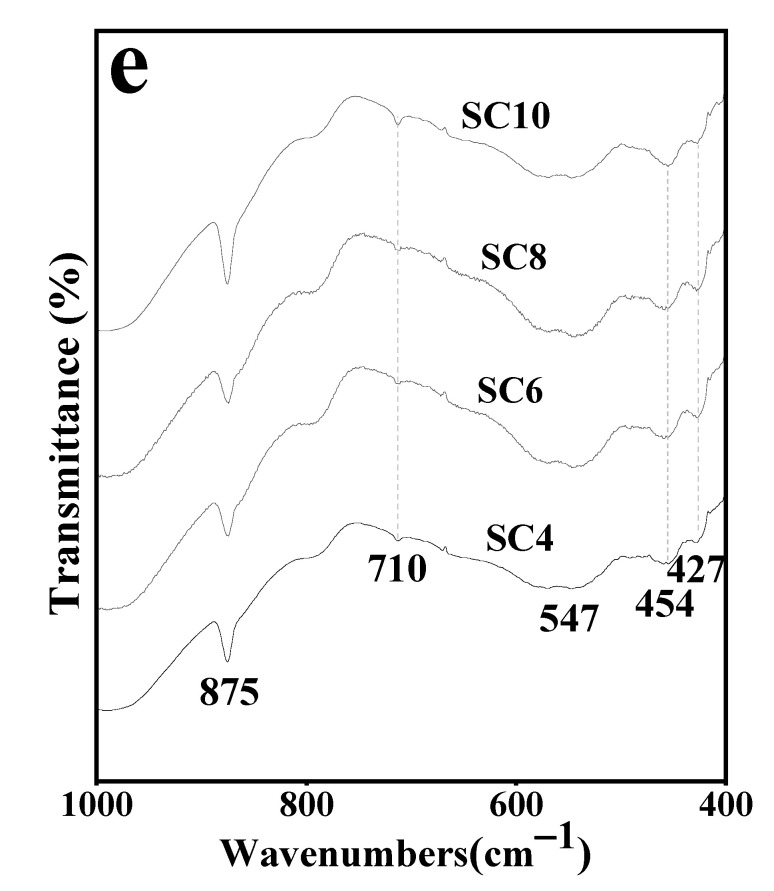
The FTIR spectra of TETS and solidified samples at 1 day (**a**), 3 days (**b**), 7 days (**c**), and 28 days (**d**), and the low wavenumber region of solidified samples at 3 days (**e**).

FTIR spectra of solidified samples with different TETS and CaO contents at different curing ages are shown in Figure 4. Absorption wavebands were classified by combining with XRD results and relevant references [40,41,42,43,44]. For TETS, the absorption peak at wavenumber ~3430 cm^−1^ was the stretching vibration peak of absorbed water molecules [45,46] and the peak at 1634 cm^−1^ was the bending vibration peak of absorbed water molecules. The peak at 989 cm^−1^ was attributed to bending vibration out of the C-O surface of CO_3_^2−^, indicating that there was carbonate in TETS. The peak at ~510 cm^−1^ was attributed to stretching vibration of the Al-OH bond in TETS [46]. The existence of the Al-OH bond provides favorable conditions for the production of FS in solidified samples of TETS.

For solidified samples of CaO-activated TETS, the absorption peak at ~3435 cm ^−1^ was attributed to stretching vibration of water molecules and the absorption peak at 1636 cm^−1^ was attributed to bending vibration of absorbed water molecules. Combining with XRD results, the absorption peak at 1428 cm^−1^ belonged to the stretching vibration peak of CO_3_^2−^ in calcite [42,43,44]. The peaks at ~990 cm^−1^ and ~875 cm^−1^ were caused by bending vibration out of the C-O surface of CO_3_^2−^ in calcite, and the peak at 710 cm^−1^ was the characteristic peak of calcite [47]. Peaks at ~547 cm^−1^, ~454 cm^−1^, and 427 cm^−1^ belonged to the Al-OH bending vibrations of FS [48].

Due to the properties of chlorine-bonded ions, the vibration peak of chloride ions was not recognized in the range of 400~4000 cm^−1^ [49]. According to FTIR and XRD spectra, calcite and FS phases were produced in solidified samples when CaO was used as the activator. Chloride binding in solidified samples was mainly attributed to chemical binding in FS.

### 3.4. Microscopic Morphology of Solidified Samples

**Figure 5:**
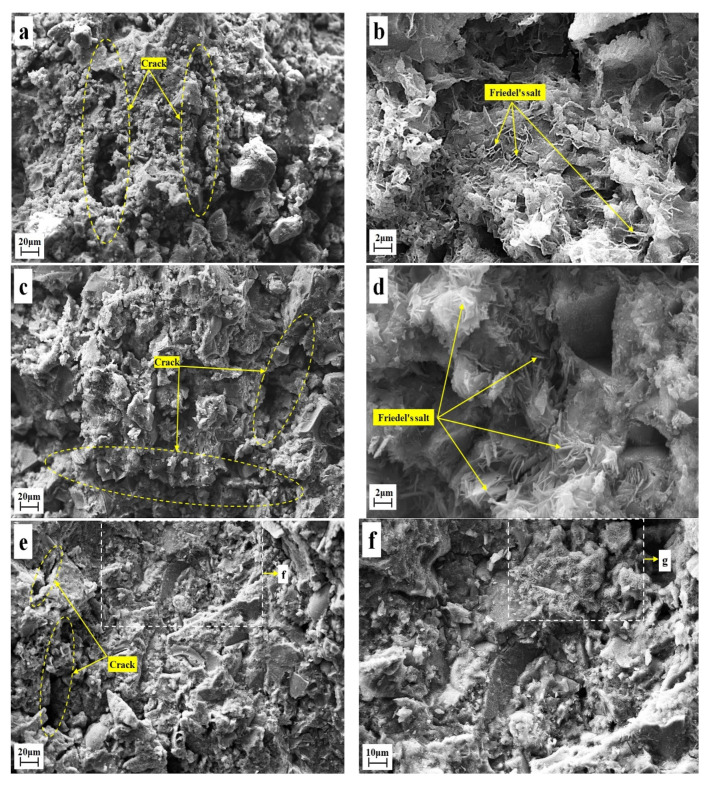

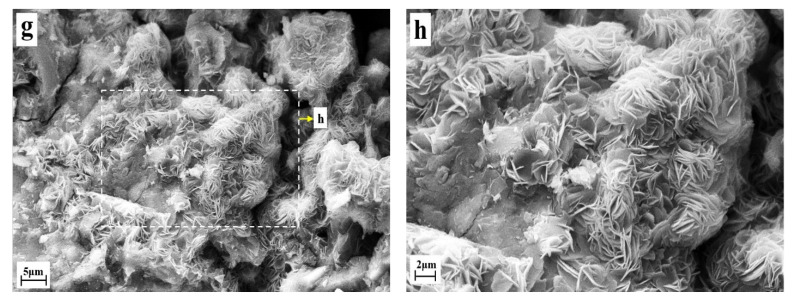
Different magnification of SEM image, SC4 sample at 28 days (**a**,**b**), SC10 sample at 7 days (**c**,**d**), SC10 sample at 28 days (**e**–**h**).

SEM images of SC4 at 28 days are shown in Figure 5a,b, SEM images of SC10 at 7 days are shown in Figure 5c,d, and SEM images of SC10 at 28 days are shown in Figure 5e–h. It can be seen from Figure 5a that there was poor binding among solidified samples’ particles in SC4 at 28 days, manifested by a loose structure and poor integrity. There were side fractures and some tabular crystals in solidified samples (Figure 5b). These crystal lamellas were relatively thin and scattered around, with underdevelopment of crystals. The integrity of SC10 at 7 days was still relatively poor (Figure 5c), but the crystal lamella was thickened, accompanied with local clusters (Figure 5d). At 28 days, the integrity of solidified samples was improved, manifested by dense structures, decreased loose particles, and few fractures (Figure 5e). Under this condition, there were abundant lamellar crystals at pores of solidified samples, which were arranged tightly. Moreover, lamellas were mutually parallel, or side surfaces were mutually offset. The lamellar thickness was smaller than 0.2 μm and lamellas were clustered at pores in the solidified samples (Figure 5e–h) [50]. The development of lamellar crystals at pores optimized the pore structure, increased the solid capacity of solidified samples, formed retardation against liquid flowing among pores, and decreased leaching of chloride ions to some extent [51,52]. Moreover, it was conducive to the strength growth of solidified samples [53,54].

**Figure 6:**
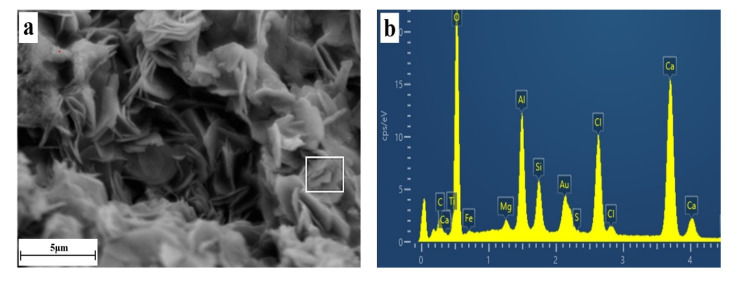

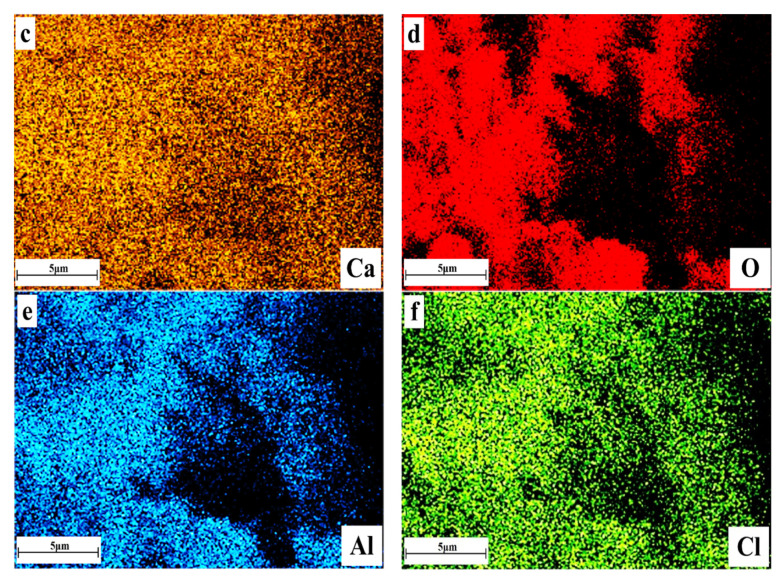
SEM (**a**) and EDS images (**b**–**f**) of the lamellar crystal region in the SC10 solidified sample at 28 days.

SEM-EDS images in lamellar crystal clustering areas in solidified samples of SC10 at 28 days are shown in Figure 6. It can be seen from Figure 6a that lamellar crystals were the major morphology of the observation area and no other crystals with significantly different shapes were discovered. The EDS curve in Figure 4d was the surface scanning composition analysis results of the chosen area in Figure 6a. Ca, O, Al, and Cl were the major components, which are also main elements of FS. Surface scanning images of Ca, O, Al, and Cl in the observation area in Figure 6a are shown in Figure 6c–f.

Samples of EDS used were not polished in the manuscript, which may be credible of the EDS mapping in a way; even so, it can still be seen that composition images of Ca, O, Al, and Cl highly conformed with the morphology in Figure 6a. According to the comparison of Figure 6c–f, distributions of Ca, O, Al, and Cl were highly correlated. Correlations among distributions of O, Al, and Cl were stronger. Combining with XRD images, the positions of appearance, and references, it can be concluded that these lamellar crystals were FS [55,56].

## 4. Conclusions

This paper mainly studied the effects of calcium oxide content and curing age on the chloride binding capacity in the solidified samples of CaO-activated TETS, and the chloride binding mechanism was revealed. The results are as follows:

(1) The solidified sample strength and chloride binding capacity of the CaO-activated TETS cementitious material were greatly affected by the CaO content and curing age. Increasing the CaO content and extending the curing age will not only help to improve the strength of the solidified sample, but also help to enhance the chloride binding capacity, especially within the curing age of 7 days. The growth rate of the strength and the chloride binding rate was the largest. The growth rate slowed down beyond 7 days. The higher the CaO content, the slower the reduction rate.
(2) The main new phase produced in the solidified sample was FS and calcite. There were no C-S-H and portlandite diffraction peaks, which were commonly found in CaO-activated GGBS cementing material. The chloride binding in the solidified sample was mainly the chemical binding by FS. The diffraction peak intensity of FS was positively correlated with the content of CaO and the curing age. However, the diffraction peak of calcite was always weak, and its strength was less affected by the CaO content and the curing age.
(3) FS is flaky and mainly existed in the pores of the solidified sample, and its production and development degree were affected by the content of CaO and the curing age. The higher the content of calcium oxide, the longer the curing age, the more FS production in the pores, and the more perfect the crystal development. FS can not only chemically bind chloride ions but can also optimize the pore structure of the solidified sample. It had two effects: chemically binding the chloride ions and improving the strength.
(4) Since the strength of the solidified sample with 6 wt% CaO reached more than 5 MPa at 7 days and more than 11 MPa at 28 days, TETS can be used for pavement base, baking-free brick, and other engineering projects with low strength requirements and not sensitive to chloride ions. This study is helpful to realize the resource utilization of TETS, and can solve the problem that TETS only be stored at present. According to the law of strength and chloride binding capacity in this paper, it is necessary to increase the CaO content to more than 10 wt%. The addition of the activated alumina component may increase the production of FS and the chloride binding capacity. All the results presented here need to be further studied.

## Figures and Tables

**Table 1:** Chemical composition and LOI of TETS (wt%).

Item	CaO	SiO_2_	Al_2_O_3_	TiO_2_	MgO	Cl	Fe_2_O_3_	SO_3_	F	MnO	LOI
Content	31.0	23.3	13.3	7.7	6.1	6.0	3.5	1.2	0.9	0.6	5.7

**Table 2:** The mix proportions for different solidified samples.

Samples	CaO/wt%	TETS/wt%	Water/Solid/wt/wt
SC4	4.0	96.0	0.40
SC6	6.0	94.0	0.40
SC8	8.0	92.0	0.40
SC10	10.0	90.0	0.40

**Table 3:** UCS and chloride binding amount of different solidified samples.

Samples	UCS (MPa)				Chloride Binding Amount (mg/g)			
	1 Day	3 Days	7 Days	28 Days	1 Day	3 Days	28 Days	7 Days
SC4	0	1.39	2.75	5.01	3.36	6.31	8.23	11.22
SC6	0.46	2.59	5.34	11.49	7.23	11.31	15.65	20.57
SC8	1.08	3.65	7.19	16.23	9.56	14.68	21.12	28.76
SC10	2.12	5.23	9.61	20.74	16.32	22.89	30.07	38.93
